# Asthma-Related School Absenteeism and School Concentration of Low-Income Students in California

**DOI:** 10.5888/pcd9.110312

**Published:** 2012-05-17

**Authors:** Ying-Ying Meng, Susan H. Babey, Joelle Wolstein

**Affiliations:** Author Affiliations: Susan H. Babey, Joelle Wolstein, University of California, Los Angeles, Center for Health Policy Research and University of California Los Angeles School of Public Health, Los Angeles, California.

## Abstract

**Introduction:**

Asthma is one of the leading causes of school absenteeism. Previous studies have shown that school absenteeism is related to family income of individual students. However, there is little research examining whether school absenteeism is related to school-level concentration of low-income students, independent of family income. The objective of this study was to examine whether the proportion of low-income students at a school was related to school absenteeism due to asthma.

**Methods:**

Using data from the 2007 California Health Interview Survey, a population-based survey of California households, we examined the association between attending schools with high concentrations of low-income students and missing school because of asthma, adjusting for demographic characteristics, asthma severity, and health insurance status. Schools with high concentrations of low-income students were identified on the basis of the percentage of students participating in the free and reduced-price meal program, data publicly available from the California Department of Education.

**Results:**

Students attending schools with the highest concentrations of low-income students were more likely to miss school because of asthma. Students from low-income families, younger students, those with more frequent asthma symptoms, or those taking prescription asthma medications also were more likely to miss school because of asthma.

**Conclusion:**

The use of school-level interventions to decrease school absenteeism due to asthma should be explored, especially in schools with high concentrations of low-income students. Potential interventions could include school-based asthma education and disease management or indoor and outdoor air pollution control.

## Introduction

Asthma is one of the most common chronic conditions among children in the United States. In 2009, an estimated 10.2 million US children (9.6%) had diagnosed asthma (1). In California in 2007, approximately 1.4 million children (15.4%) had diagnosed asthma (2). Asthma is also one of the leading causes of school absenteeism (3). In 2008, asthma accounted for an estimated 14.4 million lost days of school among children nationally (4). In California, school-aged children missed approximately 1.6 million days of school because of asthma in 2007 (2). School absences have consistently been associated with worse academic performance (5-7). Students who miss more school score lower on standardized tests in both reading and math (6,7) and are more likely to drop out before graduating from high school (5,8). Students with asthma are absent from school more than those without asthma (9). As a result, students with asthma may be more likely to experience the poor academic outcomes associated with increased absenteeism.

Students from families with lower socioeconomic status (SES) miss more school (10,11). However, few studies have examined whether school absenteeism is related to the school-level concentration of low-income students, independent of family income. Rothman found that having a higher percentage of students from low-SES families was associated with a higher school absence rate (10). If school-level factors are related to absenteeism, school-level interventions may help to reduce absenteeism. School absence due to illness such as asthma not only affects individual students but also can affect other students who attend the same schools. In California, school districts are funded on the basis of daily attendance (12,13). When students miss school, funding for their school is reduced. Because low-income school children are more likely to have severe asthma and to miss school because of asthma, schools with a large proportion of low-income students may be disproportionately affected by absences due to asthma (2). As a result, school-level interventions can affect not only the targeted students but also other students attending the same schools (14).

Examining whether school absenteeism is related to indicators of school-level disadvantage can inform the development of school-level interventions to reduce asthma-related school absences. We tested the hypothesis that children attending schools with the highest proportion of low-income students were more likely to miss school because of asthma than children attending schools with the lowest proportion of low-income students. We examined the relationship of school-level socioeconomic indicators with absenteeism while accounting for a wide range of individual-level factors including income, asthma severity, and health insurance status.

## Methods

### Data source and population

We used data from the 2007 California Health Interview Survey (CHIS), a random-digit–dialed telephone survey of households that uses a 2-stage, geographically stratified design to produce a representative sample of California’s noninstitutionalized population. Residential telephone numbers were selected from within predefined geographic areas, and respondents were then randomly selected from within sampled households. One randomly selected adult (aged ≥18 y) was interviewed in each household. In households with adolescents (aged 12–17 y), 1 adolescent was randomly selected and interviewed directly after obtaining both a parent’s permission and the adolescent’s assent. In households with children aged 0 to 11 years, 1 child was randomly selected and the adult most knowledgeable about that child was interviewed.

CHIS interviewers conducted interviews in English, Spanish, Chinese, Vietnamese, and Korean. Detailed information about the CHIS methodology is available elsewhere (15). Interview completion rates among screened households were 73.7% for children and 44.1% for adolescents (15). We analyzed responses from school-aged children (aged 4–17 y) who reported having had asthma diagnosed and who attended public school. The University of California, Los Angeles Office for the Protection of Research Subjects certified this research as exempt from review.

### Measures

For the outcome variable, we examined missing school because of asthma on the basis of responses to the following question: “During the past 12 months, how many days of school did you miss due to asthma?” Adolescents self-reported the number of school days missed, and the most knowledgeable adult reported for children. The distribution of responses to this question was highly skewed; 76% reported not missing any school days because of asthma, and only 7.6% reported missing 5 or more days of school. Thus, responses were analyzed as a dichotomous variable with the following categories: 0 days and 1 or more days. Only respondents who reported having had asthma diagnosed (in response to the question, “Has a doctor ever told you that you [or your child] have/has asthma?”) were asked about missing school because of asthma. As a result, our analysis is limited to respondents who had diagnosed asthma.

We examined the percentage of enrolled students who participated in the free or reduced-price meal program at the respondent’s school (<25%, 25%–49%, 50%–74%, ≥75%) as the primary predictor of interest. In California, a child’s family income must fall below 130% of the federal poverty guidelines ($27,564 for a family of 4 in 2007) to qualify for free meals or below 185% of the federal poverty guidelines ($39,226 for a family of 4 in 2007) to qualify for reduced-cost meals (16). This variable served as a proxy for identifying schools with high concentrations of low-income students, consistent with previous research (17,18). We obtained this information from publicly available 2007 data from the California Department of Education. We merged these data with CHIS data based on respondents’ school. Because these data are available only for public schools, we limited our analysis to students attending public schools. In addition to the meal program variable, we included covariates to adjust for individual-level family income, demographic characteristics, health insurance status, and asthma severity.

The adult respondent reported family income, and this variable was included in the analysis as a percentage of the federal poverty guidelines (≤185%, 186%–399%, and ≥400%). Analyses included the following demographic characteristics: age (4–10 y, 11–14 y, 15–17 y), sex, and race/ethnicity (white, Latino, Asian, African American, or mixed race). We also included the following indicators of asthma severity: frequency of asthma symptoms (at least monthly, less than monthly) and currently taking daily prescription medication to control asthma (yes, no). Adolescent respondents self-reported demographic characteristics and asthma indicators. The most knowledgeable adult reported this information for children. We used continuity of insurance coverage during the past year (insured all year, uninsured all or part of the year) to indicate health insurance status. The presence of secondhand smoke in the household (no smoking in house vs smoking occurs ≥1 d/wk) was assessed because this is a common asthma trigger. The adult respondent in the household reported both of these.

### Statistical analysis

We conducted logistic regression analyses to examine factors associated with missing school because of asthma. The first model examined the association of missing school because of asthma with age, sex, race/ethnicity, family income, frequency of asthma symptoms, use of daily prescription medications for asthma, insurance status, and household secondhand smoke exposure. The second model included the indicator for school-level disadvantage, proportion of low-income students, to examine the independent association of this variable with missing school because of asthma, adjusting for the covariates in the first model. The CHIS sample had 1,302 children (aged 4–17 y) with asthma who attend public school. The final analysis was limited to 1,276 children for whom data on the proportion of students at the school who participated in the meal program could be linked. Data were analyzed with SAS version 9.2 (SAS Institute, Inc, Cary, North Carolina) and SUDAAN version 10.0.1 (RTI International, Research Triangle Park, North Carolina). Analyses were weighted to be representative of the California population and adjusted for the complex survey design of CHIS.

## Results

Seventeen percent of children aged 4 to 17 who attended public school had diagnosed asthma, of whom 23% had missed at least 1 day of school because of their asthma (Table 1). The first regression model indicated that children in the lowest family income group were more likely to miss school because of asthma than children in the highest income group, while adjusting for all other covariates (Table 2). In addition, younger children, those who had more frequent asthma symptoms, or those using prescription asthma medications were more likely to miss school because of asthma.

**Table 1:** Characteristics of School-Aged Children With Diagnosed Asthma (N = 1,302), California Health Interview Survey, 2007

Characteristic	n (%)^a^
**Missed school days due to asthma**	
0	1,007 (77.2)
≥1	294 (22.8)
**Age, y**	
4–10	577 (36.3)
11–14	387 (30.0)
15–17	338 (33.7)
**Sex**	
Female	546 (41.2)
Male	756 (58.8)
**Race/ethnicity**	
White	578 (31.4)
Latino	465 (44.2)
Asian	91 (9.5)
African American	70 (9.6)
Mixed race	98 (5.4)
**How often had asthma symptoms in past year**	
Less than monthly	1,045 (81.8)
At least monthly	257 (18.2)
**Currently taking daily prescription medication to control asthma**	
Yes	304 (24.3)
No	998 (75.8)
**Insured during past year**	
Uninsured all or part of year	101 (9.9)
Insured all year	1,201 (90.2)
**Exposure to smoking at home**	
No smoking in home	1,255 (96.1)
Smoking ≥1 d/wk	47 (3.9)
**Family income, % FPG**	
<186	370 (37.0)
186–399	402 (29.0)
≥400	530 (34.0)
**Students eligible for free or reduced price meals at school, %**	
<25	409 (30.7)
25–49	373 (25.9)
50–74	277 (20.9)
≥75	217 (22.5)

Abbreviation: FPG, federal poverty guidelines.

^a^ Numbers are unweighted. Percentages are weighted to be representative of the California population. Some sample sizes may not add to total because of missing values.

**Table 2:** Odds of Missing School Because of Asthma Among School-Aged Children With Diagnosed Asthma (N = 1,276), California Health Interview Survey, 2007^a^

Characteristic	Model 1, OR (95% CI)	*P* Value	Model 2, OR (95% CI)	*P* Value
**Age y**				
4–10	1 [Reference]	NA	1 [Reference]	NA
11–14	0.47 (0.29–0.78)	.003	0.48 (0.29–0.78)	.003
15–17	0.22 (0.12–0.41)	<.001	0.24 (0.13–0.45)	<.001
**Sex**				
Male	1 [Reference]	NA	1 [Reference]	NA
Female	1.40 (0.93–2.13)	.11	1.35 (0.89–2.05)	.16
**Race/ethnicity**				
White	1 [Reference]	NA	1 [Reference]	NA
Latino	1.25 (0.79–2.00)	.35	1.09 (0.67–1.76)	.74
Asian	1.02 (0.41–2.55)	.97	1.01 (0.41–2.48)	.99
African American	1.34 (0.63–2.82)	.45	1.18 (0.55–2.51)	.67
Mixed race	0.43 (0.20–0.94)	.04	0.41 (0.17–0.96)	.04
**Insured during past year**				
Insured all year	1 [Reference]	NA	1 [Reference]	NA
Uninsured all or part of year	0.79 (0.39–1.61)	.52	0.81 (0.41–1.60)	.54
**How often had asthma symptoms in past year**				
Less than monthly	1 [Reference]	NA	1 [Reference]	NA
At least monthly	2.17 (1.31–3.60)	.003	2.29 (1.40–3.74)	.001
**Exposure to smoking at home**				
No smoking in home	1 [Reference]	NA	1 [Reference]	NA
Smoking ≥1 d/wk	2.14 (0.99–4.64)	.06	2.04 (0.92–4.50)	.08
**Currently taking prescription medication to control asthma**				
No	1 [Reference]	NA	1 [Reference]	NA
Yes	4.29 (2.77–6.67)	<.001	4.22 (2.74–6.52)	<.001
**Family income, % FPG**				
<186	2.16 (1.27–3.68)	.005	1.81 (1.05–3.12)	.03
186–399	0.99 (0.60–1.61)	.95	0.89 (0.53–1.50)	.67
≥400	1 [Reference]	NA	1 [Reference]	NA
**Students eligible for free or reduced-price meals at school, %**				
<25	NA	NA	1 [Reference]	NA
25–49	NA	NA	1.20 (0.69–2.21)	.52
50–74	NA	NA	1.31 (0.72–2.40)	.38
≥75	NA	NA	1.99 (1.07–3.71)	.03

Abbreviations: OR, odds ratio; CI, confidence interval; NA, not applicable; FPG, federal poverty guidelines.

^a^ The final analysis was limited to 1,276 children for whom data on the proportion of students at the school who participated in the meal program could be linked. Analyses adjusted for all variables displayed for each model. Model 1 did not include school-level socioeconomic status, indicated by percentage of students eligible for free or reduced-price meals. Results were weighted to be representative of the California population and were adjusted for complex survey design effects.

The results of the second model indicate that children attending schools with the highest proportion of low-income students were more likely to miss school because of asthma than children attending schools with the lowest proportion of low-income students (OR, 1.99; *P* = .03), while adjusting for age, sex, race/ethnicity, family income, insurance status, and severity of asthma (Table 2). Although the effect was somewhat attenuated, the association between family income and missing school because of asthma remained significant in model 2. As with model 1, younger children, children with more frequent asthma symptoms, or children taking prescription asthma medication also were more likely to miss school because of asthma.

## Discussion

This study found that students who attended schools with the highest concentrations of low-income students were more likely to miss school because of asthma than those at schools where the concentration of low-income students was lower. In addition to family income, the proportion of low-income students in the school is associated with asthma-related school absences after adjusting for family income, demographic characteristics, health insurance status, asthma severity indicators, and exposure to household smoke. Because California schools receive funding based on daily attendance, these findings suggest that resources allocated to schools with a large proportion of low-income students may be reduced because their children are more likely to miss school because of asthma (2,12,13). However, these findings also suggest that school-level interventions to help children manage their asthma may be explored as a strategy to decrease school absenteeism.

Our findings indicate that children from low-income families, who are younger in age, who have more frequent asthma symptoms, or who take daily prescription asthma medications were also more likely to miss school because of asthma. These findings are consistent with those of previous studies, which showed that children’s absences from school because of asthma were related to the underlying severity of their asthma (9). For instance, among children with asthma, those with more frequent asthma symptoms and those taking prescription asthma medications were more likely to miss school because of their asthma. Consistent with existing literature, our findings also showed that students from poor families were more likely to miss school than those from more affluent families (10,11).

Our analysis does not provide a direct explanation for why children attending schools with the highest concentrations of low-income students were more likely to miss school because of asthma. However, other research offers possible explanations. For example, students at disadvantaged schools may have higher exposure to indoor or outdoor asthma triggers. School absences are related to air pollution exposure (19,20). School location can determine exposure to air pollution, particularly traffic-related pollutants, and schools with a high proportion of students in the free and reduced-price meal program are more likely to be near busy roads (18). Furthermore, Simons et al found that students who attend schools with poor building conditions, such as visible mold, vermin, and poor ventilation, are more likely to miss school (21). In addition, they found that schools in lower SES districts show some of the strongest associations between poor building conditions and absenteeism. Poor building conditions may also lead to more intrusion of outdoor pollutants, such as motor vehicle exhaust (22).

School absences due to asthma can be avoided by appropriate asthma management, including appropriate use of medications and reduced exposure to triggers (23). Our results suggest that school-level interventions, especially in schools with high concentrations of low-income students, could be effective in decreasing school absenteeism due to asthma. Potential interventions could include indoor and outdoor air pollution control, for instance, locating schools, day care centers, and sports fields away from busy roadways. In 2003, California passed legislation (SB 352) that requires school districts to ensure that new schools are not built on old hazardous waste sites, hazardous release sites, or sites that contain pipelines that carry hazardous substances; in addition, school site boundaries cannot be within 500 feet from the edge of the closest traffic lane of a freeway or busy traffic corridor (24). This legislation could be a model for other states to follow.

School-based asthma education and disease management could be another effective measure to reduce absenteeism. For example, 1 study demonstrated that a comprehensive school-based asthma program led to fewer absences attributable to asthma (25). Other research suggests that children with asthma enrolled in schools without a school-based health center miss more days of school than those enrolled in schools with such a center (26). However, supplemental funding from public sources is likely needed for schools serving low-income populations to establish programs such as asthma education or school-based health centers, because these schools do not have the same capacity to raise funds from within the community as schools serving high-income populations. Many of these strategies are included in the Centers for Disease Control and Prevention’s *Strategies for Addressing Asthma Within a Coordinated School Health Program* (27).

This study has several limitations. First, the CHIS data, including our outcome indicator, school absence due to asthma, are self-reported. These data may be subject to recall bias or error in the self-reported asthma-related indicators. For instance, respondents might not be able to accurately recall the number of days of school missed due to asthma for more than the proximate past. As a result, we used the measure of 1 or more school days missed instead of actual number of school days missed, which may be less error prone. We also expect this error to be similar for those who attended schools with high or low concentrations of low-income students, that is, nondifferential with regard to SES of their schools. Second, our results are based on data from a single state, and the results may not be generalizable to all states. However, California is the most populous US state, with approximately 36.5 million residents, and its population is diverse. In addition, California often provides an early indication of health and policy trends for the rest of the nation. For example, California has more stringent state air quality standards than the nation and was the first state to regulate the siting of schools. As a result, the findings of this study could be informative for a national audience. Additionally, this study does not explain the underlying causes of school absences. Future research could examine these underlying causes by linking school-based environmental indicators, such as traffic near schools and air pollutant monitoring data, with asthma outcome data. Future research could also explore other indicators of neighborhood SES.

Although further studies are necessary to confirm the findings of this cross-sectional study and explore the underlying causes of school absences, these results suggest that school-level interventions, especially in schools with high concentrations of low-income students, should be explored as a strategy to decrease school absenteeism due to asthma. Potential interventions could include school-based asthma education and disease management and indoor and outdoor air pollution control.

## Acknowledgments

This research was supported by a grant (no. 20081418) from The California Endowment. We thank Melanie Levy for her assistance with data analysis.
